# Engineered *Zymomonas mobilis* tolerant to acetic acid and low pH via multiplex atmospheric and room temperature plasma mutagenesis

**DOI:** 10.1186/s13068-018-1348-9

**Published:** 2019-01-05

**Authors:** Bo Wu, Han Qin, Yiwei Yang, Guowei Duan, Shihui Yang, Fengxue Xin, Chunyan Zhao, Huanhuan Shao, Yanwei Wang, Qili Zhu, Furong Tan, Guoquan Hu, Mingxiong He

**Affiliations:** 1 Biomass Energy Technology Research Centre, Key Laboratory of Development and Application of Rural Renewable Energy (Ministry of Agriculture), Biogas Institute of Ministry of Agriculture, Section 4-13, Renmin Rd. South, Chengdu, 610041 China; 2 Hubei Collaborative Innovation Center for Green Transformation of Bio-resources, Environmental Microbial Technology Center of Hubei Province, Hubei Key Laboratory of Industrial Biotechnology, College of Life Sciences, Hubei University, Wuhan, 430062 China; 3 State Key Laboratory of Materials-Oriented Chemical Engineering, College of Biotechnology and Pharmaceutical Engineering, Nanjing Tech University, No. 30 Puzhu Rd, Pukou District, Nanjing, 211816 China; 4 College of Life Science, Sichuan Normal University, Section 2-1819, Chenglong Avenue, Chengdu, 610101 China

**Keywords:** Multiplex atmospheric and room temperature plasma (mARTP), Mutagenesis, Inhibitor tolerance, *Zymomonas mobilis*, Acetic acid, Low pH

## Abstract

**Background:**

Cellulosic biofuels are sustainable compared to fossil fuels. However, inhibitors, such as acetic acid generated during lignocellulose pretreatment and hydrolysis, would significantly inhibit microbial fermentation efficiency. Microbial mutants able to tolerate high concentration of acetic acid are needed urgently to alleviate this inhibition.

**Results:**

*Zymomonas mobilis* mutants AQ8-1 and AC8-9 with enhanced tolerance against acetic acid were generated via a multiplex atmospheric and room temperature plasma (mARTP) mutagenesis. The growth and ethanol productivity of AQ8-1 and AC8-9 were both improved in the presence of 5.0–8.0 g/L acetic acid. Ethanol yield reached 84% of theoretical value in the presence of 8.0 g/L acetic acid (~ pH 4.0). Furthermore, a mutant tolerant to pH 3.5, named PH1-29, was generated via the third round of ARTP mutagenesis. PH1-29 showed enhanced growth and ethanol production under both sterilized/unsterilized conditions at pH 4.0 or 3.5. Intracellular NAD levels revealed that mARTP mutants could modulate NADH/NAD^+^ ratio to respond to acetic acid and low pH stresses. Moreover, genomic re-sequencing revealed that eleven single nucleic variations (SNVs) were likely related to acetic acid and low pH tolerance. Most SNVs were targeted in regions between genes *ZMO0952* and *ZMO0956*, *ZMO0152* and *ZMO0153*, and *ZMO0373* and *ZMO0374*.

**Conclusions:**

The multiplex mutagenesis strategy mARTP was efficient for enhancing the tolerance in *Z. mobilis*. The ARTP mutants generated in this study could serve as potential cellulosic ethanol producers.

**Electronic supplementary material:**

The online version of this article (10.1186/s13068-018-1348-9) contains supplementary material, which is available to authorized users.

## Background

Cellulosic biofuels are sustainable alternative compared to fossil fuels. However, inhibitors generated during the pretreatment and hydrolysis of lignocellulosic feedstock, such as acetic acid, furfural, 5′-hydroxymethyl furfural, vanillin, and other organic acids/aldehydes/phenols, can severely inhibit cell growth and microbial fermentation efficiency [1, 2]. Among them, acetic acid is a major inhibitor that is able to destroy membrane integrity and intracellular redox homeostasis, which consequently exert osmotic stress, lower pH value, and reduce carbohydrate metabolism [3]. Series of biological, chemical, and physical methods have been developed to remove or tolerate this inhibitor from cellulosic hydrolysates [4, 5]. Among these methods, creating mutants capable of tolerating acetic acid is efficient for economic production of cellulosic biofuels.

*Zymomonas mobilis* is an attractive ethanologenic bacterium due to its high ethanol yield and high tolerance to ethanol [6, 7], uncoupling ethanol production with growth [8, 9], and wide environmental adaption under both aerobic and anaerobic conditions [10]. Recently, it has also emerged as a promising chassis for producing other bio-based products [11–13]. However, acetic acid still represents a great challenge to the conversion of lignocellulosic feedstock into bio-based chemicals by *Z. mobilis*. Rational and/or irrational methods have been widely applied in some biofuel producers, such as *S. cerevisiae* and *Escherichia coli*, to enhance tolerance to acetic acid and low pH [14, 15]. Likewise, rational modifications can help relieve acid toxicity to *Z. mobilis*. For instance, the introduction of an amino acid proton buffing peptide could improve the transient tolerance to low pH and acids in *Z. mobilis* strain CP4 [16]. The overexpression of Hfq, an RNA chaperon, in *Z. mobilis* significantly enhanced cellular tolerance against acetate, while its deletion diminished the acetate tolerance of strain Ac^R^ [17]. However, rational modification in *Z. mobilis* is still challenging due to the lack of comprehensive understanding on its functional genomics and regulatory network.

Random mutagenesis has been demonstrated as a powerful alternative to enhance tolerances in *Z. mobilis*. Recently, a mutant ZMA7-2 that is tolerant to 7 g/L acetic acid was created via adaptive laboratory evolution (ALE) [18]. The strain enabled high ethanol production using unsterilized acidic food waste hydrolysates at low pH values [19]. Alternatively, chemical mutagenesis has been widely applied in *Z. mobilis* for creating mutants. For example, mutant strain Ac^R^ tolerant to acetate [20] and flocculent mutant ZM401 (ATCC31822) with high tolerance to acetate and other inhibitors [21] were obtained by nitrosoguanidine (NTG) mutagenesis.

Atmospheric and room temperature plasma (ARTP) currently emerges as a powerful mutagenesis technique for bio-breeding [22]. It has been widely used in bacteria, fungi, and plants [23] to improve production efficiency [24–27] and/or to enhance robustness [28]. Compared to NTG or ultraviolet mutagenesis, the mutagenesis mediated by ARTP is fast, safe, and efficient with low locus bias [29]. Despite ARTP is powerful, no desirable mutant possibly could possibly be obtained under high selective pressure, such as high acetic acid level or low pH value if only one single round of ARTP mutagenesis was carried out. Recently, multi-round of ARTP mutagenesis has been applied to improve the production efficiency [30], demonstrating that it as an efficient mutagenesis strategy.

In this study, a multi-round ARTP mutagenesis named mARTP is performed with increasing selective pressures to generate mutants tolerant to 8.0 g/L acetic acid or low pH. Genomic mutation sites and NAD levels were analyzed to investigate tolerance mechanisms.

## Results and discussion

### mARTP mutagenesis in *Z. mobilis*

**Fig. 1:**
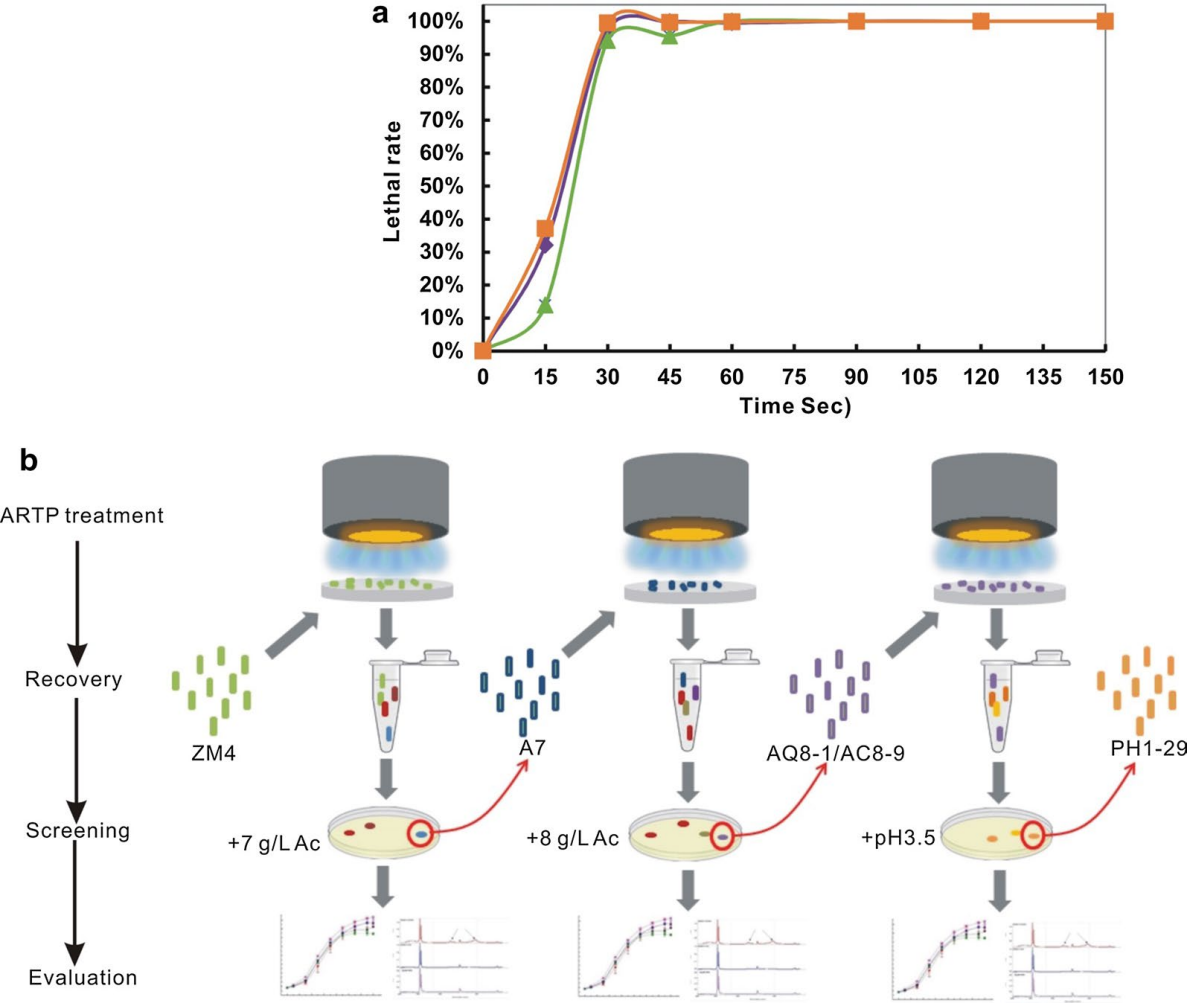
mARTP mutagenesis in *Z. mobilis*. **a** Lethal rate of ARTP mutagenesis. Results were calculated from three independent tests; **b** flow chart of mARTP mutagenesis. Details on the mutagenesis were described in “Materials and methods” and “Results”

Prior to mutagenesis, the optimal lethal rate of ARTP was determined. Results showed that mutagenesis using a radio-frequency power input at 120 W and flow rate at 10 standard liters per minute (SLM) enabled high lethal rate and stable mutation efficiency in *Z. mobilis* wild-type strain ZM4, while adjustments to 100 W and 5 or 12 SLM led to unsteady mutagenesis efficiencies (data not shown). As shown in Fig. 1a, *Z. mobilis* cells were sensitive to ARTP treatment. Treatment by ARTP for 15 s resulted in a cellular death rate between 13.8 and 37.1%, and for over 30 s resulted in no cell survival. Accordingly, *Z. mobilis* cells were treated by ARTP at 120 W, 10 SLM, and 22 °C for 30 s in this study, leading to a cellular lethal rate of 94.0–99.5%.

Previously, a *Z. mobilis* mutant against 7.0 g/L acetic acid, A7-2, was created [18], but mutants against higher concentration of acetic acid are desirable for efficient lignocellulosic ethanol production. Thus, the tolerance was further enhanced via mARTP mutagenesis in this study. After a single round of ARTP, no cells could survive when the ARTP-treated cells were immediately selected in rich medium (RM) containing 8.0 g/L acetic acid (data not shown). Such cell death could be ascribed to the dual toxicity of high acetic and low pH generated by acetic acid, as the pH value of RM containing 8.0 g/L acetic acid was decreased below 4.0 (Additional file 1: Table S1). It is hypothesized that under low pH conditions, the higher acetic acid concentration possibly led to a reduction of transmembrane pH gradient and thus to acidification of intracellular space. To solve this problem, mARTP was carried out with gradually increased selective pressures (Fig. 1b). ZM4 cells treated by the first round of ARTP were screened in RM supplemented with 7.0 g/L acetic acid. A resulting mutant A7, which had fast growth rate in the presence of 7.0 g/L acetic acid, was selected for the second round of ARTP. Consequently, two resulting mutants AQ8-1 and AC8-9 showed dramatically enhanced tolerance to 8.0 g/L acetic acid. The evidence, as shown in Additional file 1: Table S1, that pH value declined in the presence of high concentration of acetic acid suggests that the mutants’ tolerance to low pH was also improved. Hence, the third round of ARTP mutagenesis was performed using mutant AQ8-1 to screen mutants against lower pH value. A resulting mutant PH1-29 against pH 3.5 was finally obtained.

To test genetic stability, mutants were streaked on RM and grown in RM that was supplemented with acetic acid or that adjusted to pH 3.5 or 4.0. Such continuous culturing was repeated at least for three times, and there was no change of tolerance to acetic acid and pH stresses. Moreover, glycerol stocks of the mutants were stored at − 80 °C and kept stable in growth and tolerance capability so far.

By means of the mARTP mutagenesis, *Z. mobilis* mutants with “designed” tolerance capability could be generated. Thus, the mARTP mutagenesis was demonstrated to be efficient to enhance the tolerance of *Z. mobilis*.

### Improved growth and ethanol production of *Z. mobilis* against acetic acid stresses

**Fig. 2:**
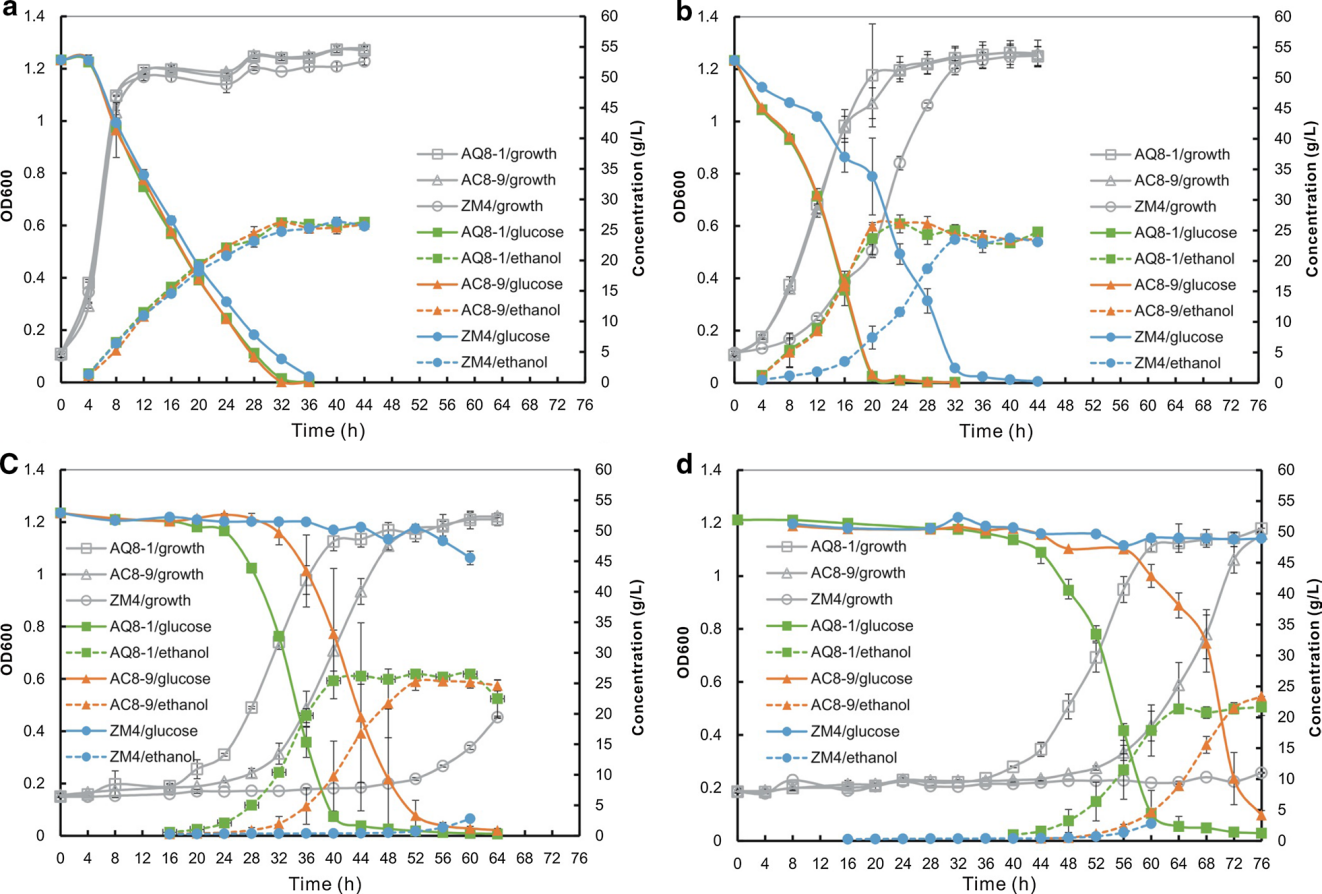
Growth and ethanol fermentation of *Z. mobilis* mutants tolerant to acetic acid. **a** RM50; **b** RM50 + 5.0 g/L acetic acid; **c** RM50 + 7.0 g/L acetic acid, and **d** RM50 + 8.0 g/L acetic acid. Growth is indicated by OD_600_ value; glucose indicates the concentration of the sugar remained in cultures; EtOH indicates the concentration of ethanol produced

As shown in Fig. 2a, b, the growth and ethanol production of mutants AQ8-1 and AC8-9 were not affected by mild acetic acid concentrations (< 5.0 g/L). In the presence of higher concentration of acetic acid (7.0 or 8.0 g/L), their growth and ethanol production were remarkably improved compared to wild-type strain, though with a longer lag phase (Fig. 2c, d and Table 1). By contrast, wild-type strain ZM4 was dramatically suppressed by over 5.0 g/L acetic acid.

**Table 1:** Ethanol production of *Z. mobilis* under acetic acid or low pH stresses

Strain	Fermentation time (h)	Glucose consumed (g/L)	Ethanol			References
			Titer (g/L)	Yield (g/g glucose consumed)	Productivity (g/L/h)	
50 g/L glucose, pH 6.0						This study
AQ8-1	28	48.06 ± 0.64	23.44 ± 1.25	0.49 ± 0.03	0.84 ± 0.04	
AC8-9	28	48.65 ± 0.84	24.45 ± 1.08	0.50 ± 0.02	0.87 ± 0.04	
PH1-29	24	49.32 ± 0.60	23.82 ± 0.44	0.48 ± 0.00	0.99 ± 0.02	
ZM4	28	45.03 ± 0.28 (< 0.01)	23.11 ± 0.06 (0.29)	0.51 ± 0.00 (0.17)	0.83 ± 0.00 (0.54)	
50 g/L glucose + 5 g/L acetic acid						
AQ8-1	20	51.77 ± 0.29	23.67 ± 2.61	0.46 ± 0.05	1.18 ± 0.13	
AC8-9	20	51.42 ± 0.40	25.67 ± 0.23	0.50 ± 0.01	1.28 ± 0.01	
ZM4	32	50.41 ± 0.54 (0.03)	23.47 ± 0.45 (0.32)	0.47 ± 0.00 (0.36)	0.73 ± 0.01 (< 0.01)	
50 g/L glucose + 7 g/L acetic acid						
AQ8-1	40	49.74 ± 0.81	25.41 ± 0.43	0.51 ± 0.01	0.64 ± 0.01	
AC8-9	56	51.24 ± 0.54	25.34 ± 0.44	0.49 ± 0.00	0.45 ± 0.01	
ZM4	60	7.35 ± 1.10 (< 0.01)	2.77 ± 0.05 (< 0.01)	0.38 ± 0.07 (0.01)	0.05 ± 0.00 (< 0.01)	
50 g/L glucose + 8 g/L acetic acid						
AQ8-1	64	49.59 ± 0.27	20.76 ± 1.16	0.42 ± 0.02	0.32 ± 0.02	
AC8-9	72	41.85 ± 4.22	21.46 ± 1.11	0.43 ± 0.00	0.30 ± 0.02	
ZM4	76	3.00 ± 0.32 (< 0.01)	0.48 ± 0.03 (< 0.01)	0.16 ± 0.01 (< 0.01)	0.01 ± 0.00 (< 0.01)	
50 g/L glucose, pH 4.0						
PH1-29^a^	24	49.60 ± 0.45	23.84 ± 0.56	0.48 ± 0.01	0.99 ± 0.02	
PH1-29^b^	32	50.23 ± 0.01	23.09 ± 1.08	0.46 ± 0.02	0.72 ± 0.03	
ZM4^a^	40	23.15 ± 1.24	11.00 ± 0.38	0.48 ± 0.01	0.27 ± 0.01	
ZM4^b^	40	8.51 ± 0.34 (< 0.01)	3.87 ± 0.06 (< 0.01)	0.46 ± 0.02 (0.28)	0.10 ± 0.00 (< 0.01)	
50 g/L glucose, pH 3.5						
PH1-29^a^	52	49.89 ± 0.16	23.04 ± 2.63	0.46 ± 0.05	0.44 ± 0.05	
PH1-29^b^	88	50.05 ± 0.02	23.93 ± 0.19	0.48 ± 0.00	0.27 ± 0.00	
ZM4^a^	60	3.17 ± 0.48 (< 0.01)	1.45 ± 0.16 (< 0.01)	0.46 ± 0.02 (0.73)	0.02 ± 0.00 (< 0.01)	
100 g/L glucose 2.1 g/L acetic acid, pH 4.22^c^						Ma et al.
A7-2	24	N/A	49.05	0.49	2.04	
100 g/L glucose 4.2 g/L acetic acid, pH 3.97^c^						
A7-2	24	N/A	48.69	0.49	2.03	
100 g/L glucose 6.3 g/L acetic acid, pH 3.85^c^						
A7-2	24	N/A	47.53	0.48	1.98	
100 g/L glucose 6.3 g/L acetic acid pH 6.0^d^						Zhao et al.
ZM401	24	99.9	48.9	0.49	2.04	
ZM4	24	99.9	46.0	0.46	1.92	
100 g/L glucose 8.4 g/L acetic acid, pH 6.0^d^						
ZM401	24	99.9	48.3	0.48	2.01	
ZM4	24	97.6	44.0	0.45	1.83	
100 g/L glucose 10.5 g/L acetic acid, pH 6.0^d^						
ZM401	36	97.4	46.2	0.47	1.28	
ZM4	48	67.5	23.9	0.35	0.50	
120 g/L glucose 16 g/L sodium acetate, pH 5.0						Liu et al.
ZMA-142^e^	56	103.9	48.0	0.46	0.86	
ZMA-142^f^	36	97.2	60.8	0.63	1.69	
ZMA-167^e^	64	100.7	44.3	0.44	0.69	
ZMA-167^f^	38	86.6	47.4	0.55	1.25	

*p* values calculated by one-way ANOVA are listed in brackets. Three repeats were performed for each strain, and error bars indicated standard deviation

^a^Sterilized and ^b^unsterilized RM containing 50 g/L glucose

^c^Concentrations of acetic acid were evaluated based on that original data (0.2%, 0.4%, and 0.6% (v/v) acetic acid was added, respectively)

^d^Adding 2 N KOH to adjust pH value to 6.0

^e^Seed culture in RM; fermentation in RM with 195 mM sodium acetate

^f^Seed culture in RM with 146 mM sodium acetate; fermentation in RM with 195 mM sodium acetate

Although AQ8-1 and AC8-9 were generated in the same mutagenesis process, they still showed difference in acetic acid tolerance. There was no remarkable difference in growth, glucose consumption, and ethanol production between both mutants below 5.0 g/L acetic acid (Fig. 2a, b). However, AQ8-1 grew faster than AC8-9 in the presence of 7.0 g/L or 8.0 g/L acetic acid (Fig. 2c, d), consequently with higher ethanol productivities (Table 1).

In addition, the tolerance of AQ8-1 and AC8-9 against mixtures of acetic acid and furfural, which are produced in cellulosic hydrolysates and are two major inhibitors to *Z. mobilis* [31, 32], was also investigated. So far, the reported highest concentrations of acetic acid and furfural *Z. mobilis* could tolerate were 7.0 g/L and 3.0 g/L, respectively [18]. Therefore, the concentration of two inhibitors in mixtures arranged from 1.0 to 7.0 g/L and 1.0 to 3.0 g/L, respectively. The mutants did not show obvious advantages over ZM4 under mixed stresses (Additional file 1: Figure S1), suggesting that the tolerance of both mutants to furfural was not enhanced. In the future, additional work is needed to improve the tolerance of *Z. mobilis* to multiple stresses via ARTP and/or other techniques.

### Improved growth and ethanol production of *Z. mobilis* at low pH

AQ8-1 and AC8-9 were conferred with enhanced tolerance to low pH as well as acetic acid since the pH value was reduced below 4.0 with the addition of 8 g/L acetic acid (Additional file 1: Table S1). Low pH circumstance can effectively prevent microbial contamination during fermentation, thus can realize the fermentation under non-aseptic conditions [33]. In this study, a mutant pH1-29 against low pH was created using AQ8-1 as the parental strain through mARTP mutagenesis.

**Fig. 3:**
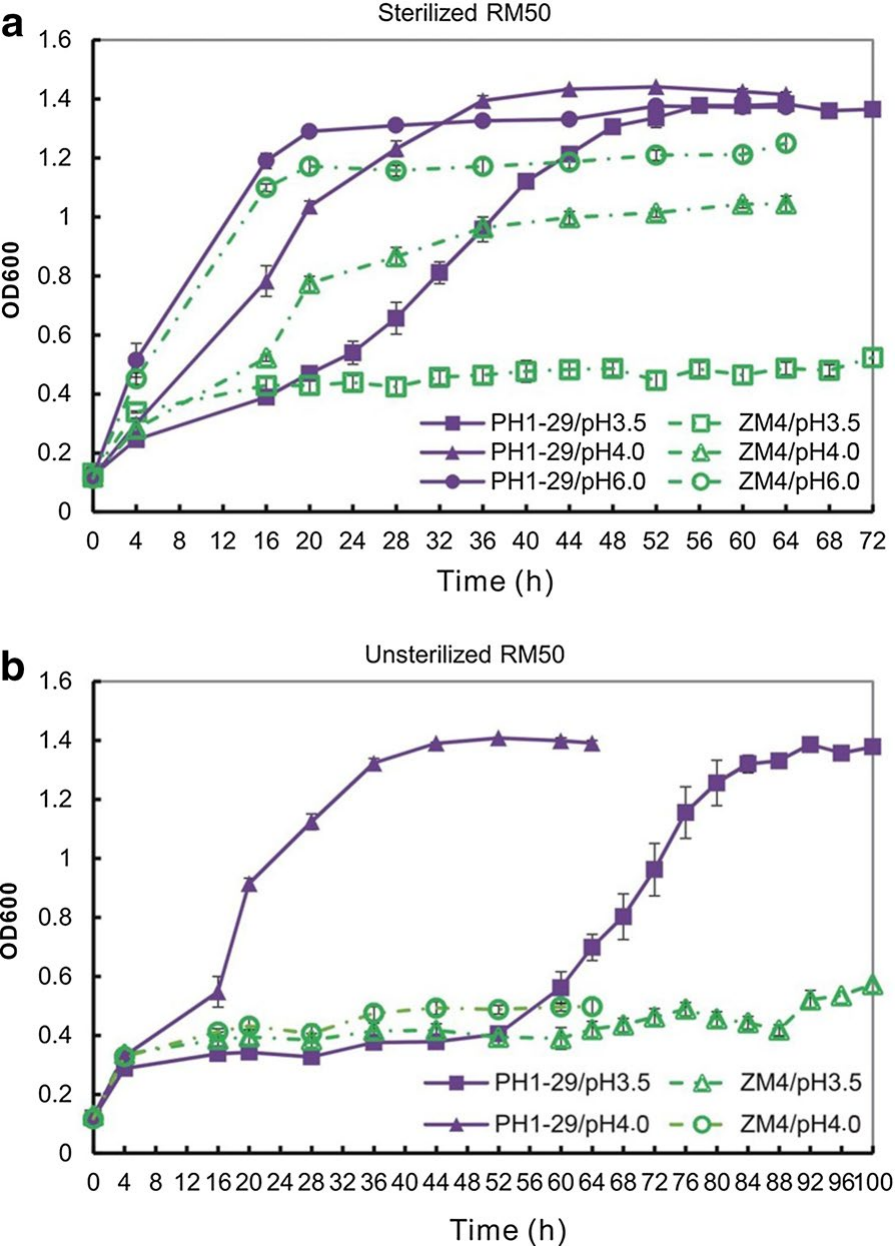
Growth curve of low pH-tolerant mutant PH1-29 in **a** sterilized and **b** unsterilized RM

The growth of PH1-29 at low pH was enhanced as compared to ZM4 (Fig. 3a). The ethanol production was not inhibited at pH 4.0, while the productivity reduced to 0.5 g/L/h at pH 3.5, nearly by a half of that at pH 6.0. In contrast, ZM4 was severely inhibited at low pH values. It grew poorly at pH 4.0 and hardly survived at pH 3.5 (Fig. 3a), resulting in severe suppression of ethanol production at low pH values (Table 1).

Furthermore, the growth and ethanol production of PH1-29 were also improved under unsterilized conditions. The growth curve of PH1-29 under unsterilized condition showed similar to that under sterilized conditions but with longer lag phase of growth at pH 3.5 (Fig. 3b). Its ethanol yields kept stable under both sterilized and unsterilized cultures (Table 1), but ethanol productivity was declined to 0.3 g/L/h at pH 3.5. These evidences showed that it has stronger tolerance to low pH than the starting strain under non-aseptic conditions.

### Comparison of ethanol production under acetic acid stress conditions

Mutants AQ8-1, AC8-9, and PH1-29 showed great potential to produce ethanol under unsterilized and low pH conditions. The ethanol productivities of AQ8-1 and AC8-9 reached at 1.3 g/L/h and 1.4 g/L/h, respectively, in the presence of 5 g/L acetic acid (pH 4.0). Even the addition of 8.0 g/L acetic acid did not dramatically affect their growth and ethanol yields (Fig. 2d), only with the decline of ethanol productivities to 0.4 and 0.3 g/L/h, respectively (Table 1). In comparison, another acetic acid-tolerant strain A7-2 that was mentioned above was able to produce ethanol at the productivity of 2.0 g/L/h in the presence of 2.1 to 6.3 g/L acetic acid with pH values ranging from 4.2 to 3.8 (Table 1). Further optimization of fermentation process will help improve the ethanol productivity of the mutant strains.

A flocculating mutant ZM401 showed strong tolerance to acetic acid. Its ethanol productivity reached at 2.0 g/L/h in the presence of 8.4 g/L acetic acid. Even in the presence of 10.5 g/L acetic acid, its ethanol productivity was far higher than that of AQ8-1 and AC8-9 [34] (Table 1). In that work, pH value was maintained at 6.0. However, the pH value was uncontrolled in this study. As mentioned above, dual effects of acetic ion and low pH resulted from acetic acid could partially explain the low productivity of AQ8-1 and AC8-9 in this study.

The tolerance of AQ8-1 and AC8-9 against sodium acetates was also tested, but there was no advantage over ZM4 (data not shown), suggesting the different effects of acetic acid and acetate salt on cells. In the future, the performance of the mutant generated, AQ8-1, AC8-9, and PH1-29, still remains to improve in the future combining with ALE [35], genome shuffling [36], or metabolic engineering [37, 38].

### Mutation analysis

**Fig. 4:**
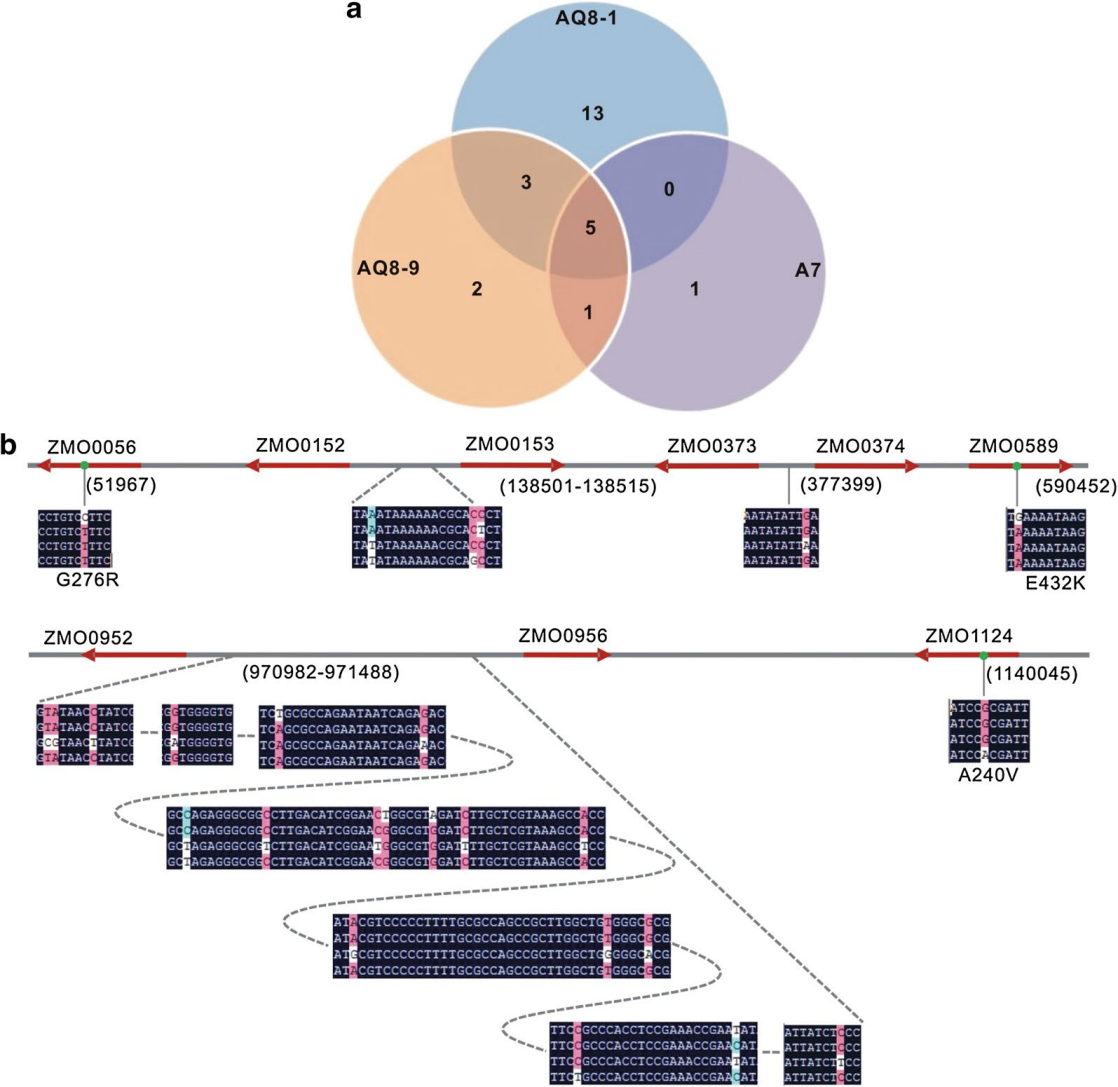
Variations in the ARTP mutants tolerant to acetic acid. **a** Venn gram of SNVs in A7, AQ8-1, and AC8-9. **b** “Hot spots” of mutagenesis loci in the ARTP mutants. Gene names are shown above the lines, arrows indicate transcriptional directions. Variations are highlighted in nucleotide rows which from top to bottom represent ZM4, A7, AQ8-1, and AC8-9, respectively

Mutant genomes were sequenced to investigate genetic changes that were caused by ARTP mutagenesis. There were dozens of single-nucleotide variations (SNVs) identified in A7, AQ8-1, AC8-9, and PH1-29, respectively (Fig. 4a, Tables 2, 3). It was found that more SNVs were created with the increase of ARTP treating cycles. Five same SNVs were detected in A7, AQ8-1, and AC8-9, two of which were located in coding sequence (CDS) and three in intergenic regions. SNVs in CDS regions led to two nonsynonymous amino acid (AA) changes in glutamine–fructose-6-phosphate aminotransferase encoded by gene *ZMO0056*, and RadA encoded by *ZMO0589*, respectively. Intergenic SNVs detected in all mutants were targeted in regions between gene *ZMO0952* and *ZMO0956*. In addition, more unique SNVs were detected in AQ8-1 than in A7 and AC8-9, being targeted in intergenic regions between *ZMO0152* and *ZMO0153*, *ZMO0373* and *ZMO0374*, and *ZMO0952* and *ZMO0956* (Fig. 4, Tables 2, 3). Variation loci in mutant PH1-29 were quite similar to that in AQ8-1 except with two distinct SNVs at locus 703683 and 971488. All SNV sites are listed in Tables 2, 3.

**Table 2:** SNVs by ARTP mutagenesis in CDS

	Locus	Ref	SNV	AA change	AQ 8-1	AC 8-9	A7	PH 1-29	A7-2	Gene/product
1	51967	C	T	G276R	+	+	+	+	+	*ZMO0056*/glutamine–fructose-6-phosphate aminotransferase
2	122147	G	A	D104N	−	−	−	−	+	*ZMO0133*/outer membrane-associated acid tolerance proteins
3	122153	C	G	Q106E	−	−	−	−	+	
4	122161	G	C	Syn.	−	−	−	−	+	
5	122169	T	G	F111L	−	−	−	−	+	
6	122170	T	G	F111L	−	−	−	−	+	
7	122172	T	A	F112Y	−	−	−	−	+	
8	122179	A	G	Syn.	−	−	−	−	+	
9	122195	T	A	L120M	−	−	−	−	+	
10	122196	T	A	L120X	−	−	−	−	+	
11	122197	G	T	L120F	−	−	−	−	+	
12	122201	G	T	E122X	−	−	−	−	+	
13	122202	A	C	E122A	−	−	−	−	+	
14	122214	G	A	S126N	−	−	−	−	+	
15	122235	C	A	T133N	−	−	−	−	+	
25	590452	G	A	E432K	+	+	+	+	+	*ZMO0589*/DNA repair protein RadA
26	703683	A	G	C141R	−	−	−	+	−	*ZMO0708*/phosphoribosyl-glycinamide formyl transferase
27	1140045	G	A	A240V	−	+	−	−	−	*ZMO0843*/arginine–tRNA ligase

Syn.: synonymous, variation in nucleotide led to no amino acid change. +/− indicate the presence/absence of variation in the genome, respectively

**Table 3:** SNVs by ARTP mutagenesis in intergenic regions

	Locus	Ref	SNV	AQ8-1	AC8-9	A7	PH 1-29	A7-2	Gene/product
1	138501	A	T	+	+	−	+	+	*ZMO0152*/pyruvate kinase *ZMO0153*/YebC/Pmp R family DNA binding transcriptional factor
2	138514	C	G	−	+	−	−	+	
3	138515	C	T	−	−	+	−	+	
4	377399	G	A	+	−	−	−	−	*ZMO0372*/AsnC family transcriptional regulator *ZMO0373*/hypothetical protein *ZMO0374*/levansucrase
5	970982	T	C	+	−	−	+	+	*ZMO0952*/(tRNA cytidine-2′-*O*) methyltransferase *ZMO0956*/ubiquinol–cytochrome C reductase
6	970983	A	G	+	−	−	+	+	
7	970988	C	T	+	−	−	+	+	
8	971023	G	A	+	−	−	+	+	
9	971059	T	A	+	+	+	+	+	
10	971077	G	A	+	−	−	+	+	
11	971114	C	T	+	+	−	+	+	
12	971128	C	T	+	−	−	+	+	
13	971129	T	G	+	+	+	+	+	
14	971135	A	G	+	+	+	+	+	
15	971139	C	T	+	−	−	+	+	
16	971154	A	T	+	−	−	+	+	
17	971183	A	G	+	−	−	+	+	
18	971214	T	G	+	−	−	+	+	
19	971219	G	A	+	−	−	+	+	
20	971312	C	T	+	+	−	+	+	
21	971332	T	C	–	+	+	+	+	
22	971488	C	T	+	−	−	+	+	

+/− indicate the presence/absence of variation in the genome, respectively

Many SNVs were detected in intergenic regions between *ZMO0152* and *ZMO0153*, and between *ZMO0952* and ZMO0956 in acetic acid-tolerant mutants A7, AQ8-1, AC8-9, and A7-2 (Fig. 4b). Considering that mutant A7-2 and mutants A7, AQ8-1 and AC8-9 were created by different means, these variation regions are likely to function in the response/tolerance to acetic acid in *Z. mobilis*. The gene *ZMO0152* encodes a pyruvate kinase (PYK), whose upstream region was reported to regulate the acid tolerance. For example, a PYK of *Lactobacillus bulgaricus* was regulated by the catabolite control protein A by binding to the kinase-encoding gene *pyk* in the presence of acid stress [39]. The differential proteomic profiling of *Streptococcus mutans* also revealed that the up-regulated PYK enhanced tolerance against acid stress [40]. Putative A/T-rich binding elements, for example, (5-TGTAAGCCCTAACA-3) upstream the –35 region, were summarized in the above literature. In ARTP mutants created in this study, variations were detected in A/T-rich regions similar to these binding elements (data not shown). Thus, a similar regulation likely exists in these ARTP mutants. Moreover, 18 variations were located in the intergenic region of *ZMO0952* and *ZMO0956*. The former gene encodes a tRNA (cytidine/uridine-2′-*O*-)-methyltransferase and the latter encodes a Fe–S subunit of ubiquinol–cytochrome *c* reductase. It was still unknown why so many SNVs were located in the region, and the function of this region in acetic acid tolerance remains to investigate in the future.

ARTP mutagenesis also revealed that several genes likely contributed to acid tolerance/response. The gene *ZMO0056* encodes a glutamine–fructose-6-phosphate aminotransferase that was reported to be critical for cells against organic acid stress [41, 42]. Such proteins contain a conserved GlmS superfamily domain that is involved in the cell wall/membrane biosynthesis [43]. Even though there is no direct evidence demonstrating its role in acid tolerance, cell wall/membrane is essential for keeping cellular integrity. The gene *ZMO0859* encodes a DNA repair protein RadA, which is essential for the survival and genetic stability when cells suffered from the acid stress [44, 45]. An alanine at position 272 of RadA was replaced with a valine in ARTP mutants as well as A7-2, but the mechanism on how such a mutation can influence gene expression and regulation is yet to be investigated in the future. A unique SNV at locus 377399 is present only in AQ8-1. It is likely to regulate the expression of genes *ZMO0372*, *ZMO0373,* and *ZMO0374* (Fig. 4 and Tables 2, 3). The gene *ZMO0372* encodes a putative leucine responsive regulatory protein/regulator of asparigine synthase C (Lrp/AsnC) transcriptional regulator which is involved in amino acid transport metabolism and cell replication [46]. The gene *ZM0374* encodes a levansucrase, a functional protein possibly involved in tolerance to multiple stresses [47]. Even though their functions in tolerating acetic acid and low pH remain unknown, the microarray revealed the down-regulation of *ZMO0372* and *ZMO0373* under acetate stress in *Z. mobilis* strain 8b [32]. Therefore, this region likely plays an important role in regulating acetic acid tolerance.

On the other hand, based on variation sites in the mutants generated by different means, we propose that mechanisms of *Z. mobilis* responding to acetic acid and to acetate salts are different. The same SNVs that were detected in both *Z. mobilis* acetate-tolerant mutants 8b and Ac^R^ were barely detected in acetic acid-tolerating mutants A7-2 [18], A7, AQ8-1 and AC8-9, and vice versa (Tables 2, 3).

### Intracellular NAD levels

**Fig. 5:**
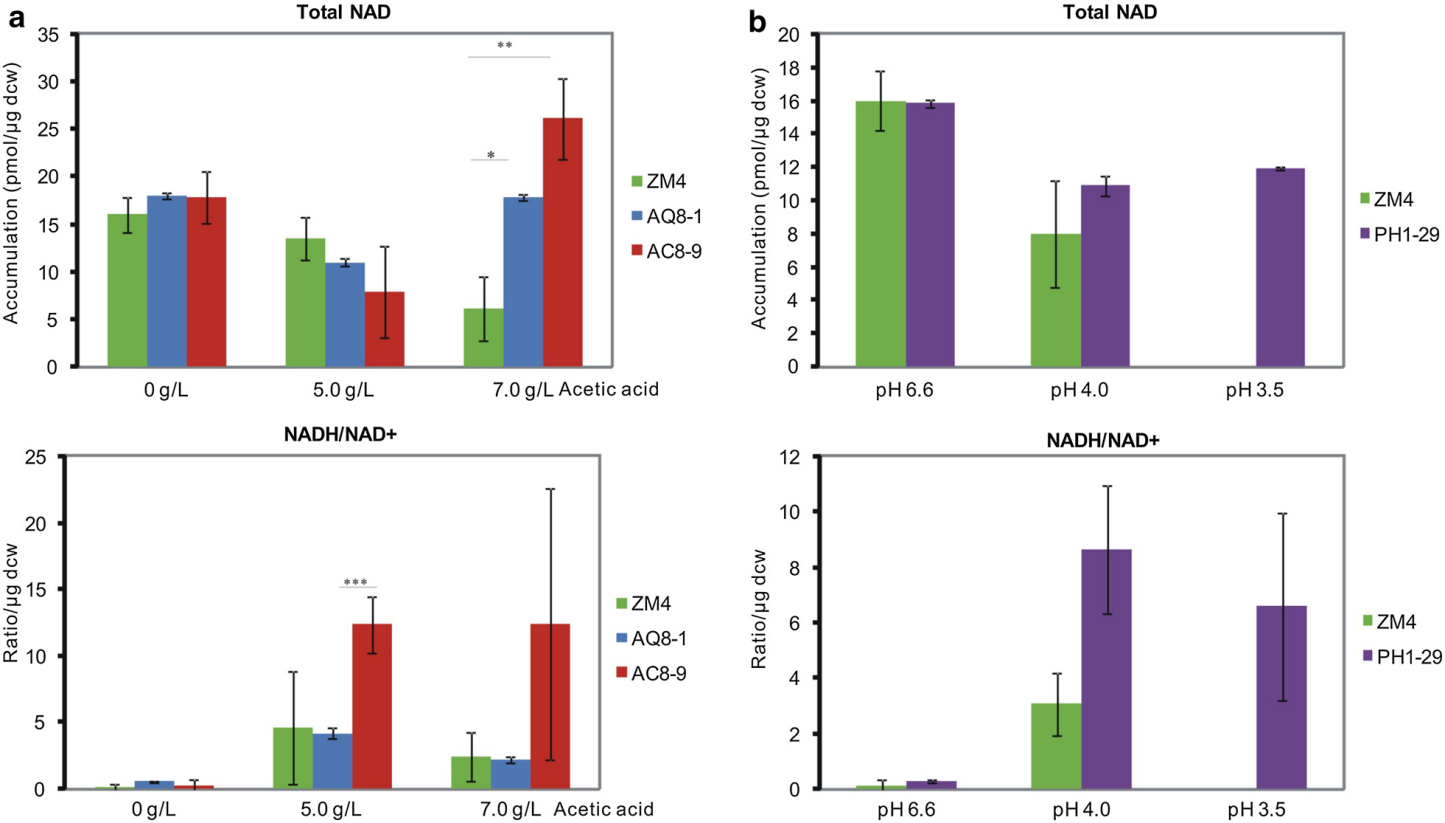
Accumulation of intracellular NAD levels under **a** acetic acid conditions, and **b** low pH conditions. Total NAD comprises NADH and NAD^+^; NADH/NAD^+^ = NADH/(total NAD − NADH). Two repeats were performed for each strain, and error bars indicate standard deviation. The change of total NAD or NADH/NAD^+^ per microgram dry cell weight (DCW) between two time points indicated the accumulation of NAD or NADH/NAD^+^. DCW was calculated from OD _600 nm_ (1OD_600_ = 0.323 gDCW/L). Asterisks above bars indicate *t* test significance between mutants and ZM4.*p = 0.0415; **p = 0.0384; ***p = 0.0326

It has been reported that intracellular NADH/NAD^+^ ratio increased in *Z. mobilis* as well as *S. cerevisiae* under stress conditions [48, 49]. Thus, the accumulation of total nicotinamide adenine dinucleotide (NAD) as well as NADH/NAD^+^ ratio was measured to investigate cellular responses to acetic acid and low pH. Total NAD and NADH/NAD^+^ remained low in RM without stresses (Fig. 5), which agreed with the reports mentioned above. Cellular total NAD levels of AQ8-1 and AQ8-9 were comparable to that of ZM4 in RM or RM with 5.0 g/L acetic acid, but were significantly higher than ZM4 in the presence of 7.0 g/L acetic acid. However, NADH/NAD^+^ ratio displayed different trends between strains. The ratio remained low in ZM4 and AQ8-1 while obviously increased in AC8-9 under acetic acid stress conditions (Fig. 5a). Similarly, the accumulation and a high NADH/NAD^+^ ratio were detected in PH1-29 under low pH stress conditions (Fig. 5b).

Under salinity stress conditions, the increase of NADH/NAD^+^ was reported in *Z. mobilis*. The respiratory chain played important roles in maintaining the redox balance by oxidizing NADH to NAD^+^ [48]. The authors suggested that NADH/NAD^+^ ratio is likely responsible for a wide stress tolerance. In the present study, many SNVs nearby the gene encoding Fe–S subunit of ubiquinol–cytochrome *c* reductase, which is an important member in respiratory chain, were detected in mutants (Fig. 4 and Table 3). Therefore, it was speculated that NADH/NAD^+^ under acetic acid and low pH stress conditions was also regulated by the similar mechanism. This can explain that a relatively low NADH/NAD^+^ of AQ8-1 was maintained under acetic acid conditions.

In contrast, the ratio in AC8-9 and PH1-29 was relatively high under stress conditions. Although the growth and ethanol productivities of AC8-9 under acetic acid stress conditions were lower than AQ8-1 (Fig. 2 and Table 1). It still enabled relatively high growth and ethanol production under stress conditions. Similar growth and ethanol production were detected in PH1-29 (Fig. 3 and Table 1). Since a high NADH or NADH/NAD^+^ level is toxic to cells, these results suggested that enhanced ability of maintaining integrity of cellular membrane and resisting high level of protons represents an alternative tolerance mechanism in AC8-9 and PH1-29.

## Conclusion

Mutant strains able to tolerate 8.0 g/L acetic acid and pH 4.0 and 3.5 were generated via the mARTP mutagenesis in this study. Therefore, the mARTP mutagenesis is efficient to create *Z. mobilis* mutants with enhanced tolerance to acetic acid and low pH. These mARTP mutants could modulate NADH/NAD^+^ ratio to respond to stress. Genome re-sequencing revealed the “hot spots” which are likely involved in tolerance to acetic acid and pH. Mutant strains generated in this study will not only serve as potential bioethanol producers, but also help understand the stress response and regulation in *Z. mobilis*.

## Materials and methods

### Cell growth

A single colony of *Z. mobilis* strain ZM4 was inoculated in 10 mL of RM (20.0 g/L glucose, 10.0 g/L yeast extract, and 2.0 g/L KH_2_PO_4_) and grown overnight at 30 °C without shaking. Cell pellets were harvested from 8 mL of RM cultures by centrifuging at 3000 rpm and 4 °C and then inoculated in 80 mL of RM that were supplemented with acetic acid or that at low pH values. Thirty-five or forty-four microliters of concentrated H_2_SO_4_ were added in 100 mL of RM to adjust pH values to 4.0 or 3.5. For tolerance tests, glucose concentration in the media was 50.0 g/L (RM50); otherwise, RM was used for the growth of *Z. mobilis*.

### mARTP mutagenesis

ZM4 cells (10^6^ to 10^8^ cells/mL) were centrifuged at 3000 rpm and 4 °C and then suspended in 0.9% NaCl solution. Ten microliters of cell aliquot were spread on a carrier plate and treated for 15 to 150 s in a Type M ARTP Mutagenesis Bio-breeding Machine (Wuxi Tmaxtree Biotechnology Co., Ltd., China). Gas helium (He) of high purity (99.999%) served as working gas at the flow rate of 10–20 SLM. The radio-frequency power input was set at 100 or 120 W and jet temperature was controlled at 22 °C using a water cycler. The treated cells were suspended in 1 mL of RM, followed by spreading on RM supplemented with 7.0 g/L acetic acid. Those resulting colonies able to grow with higher OD_600nm_ values in RM with 7.0 g/L acetic acid were then subjected to a second ARTP mutagenesis. The treated cells were screened in RM containing 8.0 g/L acetic acid. To select low pH-tolerant mutants, a mutant able to tolerate 8.0 g/L acetic acid was subjected to another round of ARTP mutagenesis, followed by screening on RM at pH 3.5. To test genetic stability, the mutants were continuously cultured in RM with acetic acid or RM at pH 4.0 or 3.5 for at least three times, and their glycerol stocks were stored at − 80 °C and their growth and tolerance were checked every 3–4 weeks.

### Genomic DNA isolation

Cells from 3 to 5 mL of *Z. mobilis* overnight cultures were harvested by centrifuging at 13,000 rpm for isolating genomic DNAs (gDNAs) using a Bacterial DNA Kit (Omega Biotek, USA). The concentration of gDNAs was determined using a Qubit 3 Fluorometer (ThermoFisher, USA). The quality of gDNAs was checked via gel electrophoresis (0.7% agarose, 12 V/cm, 60–70 min).

### Amplification of 16S rRNA gene by PCR

To ensure that the mutants are affiliated with *Z. mobilis*, 16S rRNA gene was amplified by polymerase chain reaction (PCR). Fresh cells harvested from 50 μL of overnight cultures were washed and re-suspended in 10 μL of ddH_2_O. One microliter of the suspension were used for PCR. 16S rRNA genes were amplified using general primer set 15F (AGAGTTTGATCCTGGCTCAG)/1492R (TACGGYTACCTTGTTACGACTT) and KOD-Plus-Neo polymerase (TOYOBO, Japan). PCRs were performed with a program as follows: predenaturing at 95 °C for 5 min, 30 cycles of denaturing at 98 °C for 10 s, annealing at 60 °C for 30 s, and extending at 68 °C for 1 min. Amplicons were sequenced in GENEWIZ Inc. (Suzhou, China).

### Genome re-sequencing

Genomic DNAs of the mutants were sequenced to investigate mutations. Briefly, libraries of gDNA were constructed and then re-sequenced using a Illumina HiSeq instrument (Illumina, San Diego, CA, USA). After the removal of adaptors, PCR primers, the content of *N* bases > 10%, and bases of quality lower than 20, clean data to the reference genome of strain ZM4 (GenBank No. NC_006526.2) were mapped. Annotation for potential SNVs was performed by Annovar (V21 Feb 2013). The sequencing was completed by GenWize Inc. (Suzhou, China).

### Analytical methods

Glucose and ethanol were calculated using high-performance liquid chromatography (HPLC; Agilent 1200), according to the description by You et al. [50]. Briefly, an HPX-87H ion exclusion column (Bio-Rad Aminex) was used and 5 mM H_2_SO_4_ worked as the mobile phase. The chromatographic system was run at a flow rate of 0.6 mL/min and 35 °C. The injection volume was set to 20 μL.

Cells were harvested for assaying intracellular NAD using a NAD/NADH Assay Kit (ab65348, Abcam Inc.). Briefly, one milliliter of fresh cells was harvested by centrifuging at 4000 rpm and 4 °C for 10 min. After washing with cold phosphate buffer solution (PBS), cells were extracted using NADH/NAD extraction buffer via two freeze/thaw cycles of 20 min at − 80 °C followed by 10 min at room temperature (RT). After a short vortex, supernatants were collected by centrifuging at 4 °C for 5 min. For measuring total NAD, 50 μL of extracted aliquots and 100 μL of reaction mix were added in each well of a 96-well microplate, incubated at RT for 5 min. 10 μL of NADH developer were added in each well and mixed, and OD_450nm_ values were then read using a microplate reader (Spectro MAX190, Molecular Devices, USA). For measuring NADH, extracted aliquots were heated to decompose NAD^+^ before blending with reaction mix.

## Additional file

**Additional file 1: Table S1.** pH values of RM supplemented with acetic acid or sodium acetate. **Figure S1.** Tolerance of AQ8-1, AC8-9 and ZM4 to mixture of acetic acid and furfural. “A” indicates acetic acid and “F” indicates furfural. The number after A or F indicates the concentration of corresponding inhibitor. The concentrations of acetic acid ranged from 1.0 to 7.0 g/L, and furfural from 1.0 to 3.0 g/L in the mixture. OD_600 nm_ values were measured at stationery phase.

## Abbreviations

ARTP: atmospheric and room temperature plasma
mARTP: multiplex atmospheric and room temperature plasma
ALE: adaptive laboratory evolution
NTG: nitrosoguanidine
RM: rich medium
OD: optical density
CDS: coding sequence
SNV: single-nucleotide variation
PYK: pyruvate kinase
G: guanine
C: cytosine
AA: amino acid
PCR: polymerase chain reaction
HPLC: high-performance liquid chromatography
RT: room temperature
gDNA: genomic DNA
NAD: nicotinamide adenine dinucleotide
NAD^+^: oxidative NAD
NADH: reductive NAD
SLM: standard liter per minute
PBS: phosphate buffer solution
DCW: dry cell weight
