# Supplementary material for: Engineered Zymomonas mobilis tolerant to acetic acid and low pH via multiplex atmospheric and room temperature plasma mutagenesis
Source: Biotechnol Biofuels. 2019 Jan 5;12:10. doi: 10.1186/s13068-018-1348-9 (PMC6321654; doi:10.1186/s13068-018-1348-9)
Supplement: Supplementary file 1 — Additional file 1: Table S1. pH values of RM supplemented with acetic acid or sodium acetate. Figure S1. Tolerance of AQ8-1, AC8-9 and ZM4 to mixture of acetic acid and furfural. “A” indicates acetic acid and “F” indicates furfural. The number after A or F indicates the concentration of corresponding inhibitor. The concentrations of acetic acid ranged from 1.0 to 7.0 g/L, and furfural from 1.0 to 3.0 g/L in the mixture. OD600 nm values were measured at stationery phase. [file 13068_2018_1348_MOESM1_ESM.docx]

Additional file

Engineered *Zymomonasmobilis* tolerant to acetic acid and low pH via multiplex atmospheric and room temperature plasma mutagenesis

Bo Wu^1†^, Han Qin^1†^, Yiwei Yang^1^, Guowei Duan^1^,Shihui Yang^2^, Fengxue Xin^3^, Chunyan Zhao^4^, Huanhuan Shao^4^, Yanwei Wang^1^, Qili Zhu^1^, Furong Tan^1^,Guoquan Hu^1^ and Mingxiong He^1^*

^1^ Biomass Energy Technology Research Centre, Key Laboratory of Development and Application of Rural Renewable Energy (Ministry of Agriculture), Biogas Institute of Ministry of Agriculture, Section 4-13, Renmin Rd. South, Chengdu 610041, China

^2^ Hubei Collaborative Innovation Center for Green Transformation of Bio-resources, Environmental Microbial Technology Center of Hubei Province, Hubei Key Laboratory of Industrial Biotechnology, College of Life Sciences, Hubei University, Wuhan, 430062, China

^3^ State Key Laboratory of Materials‑Oriented Chemical Engineering,College of Biotechnology and Pharmaceutical Engineering, Nanjing TechUniversity, No. 30 Puzhu Rd, Pukou District Nanjing, Nanjing 211816, China

^4^ College of Life Science, Sichuan Normal University, Section 2-1819, Chenglong Avenue, Chengdu 610101, China

Table S1 pH values of RM supplemented with acetic acid or sodium acetate

| **Medium** | **pH value** |
| --- | --- |
| RM | 6.60 |
| RM+ 1 g/L acetic acid | 4.90 |
| RM+ 2 g/L acetic acid | 4.48 |
| RM+ 3 g/L acetic acid | 4.28 |
| RM+ 4 g/L acetic acid | 4.15 |
| RM+ 5 g/L acetic acid | 4.05 |
| RM+ 6 g/L acetic acid | 3.98 |
| RM+ 7 g/L acetic acid | 3.92 |
| RM+ 8 g/L acetic acid | 3.86 |
| RM+ 9 g/L acetic acid | 3.81 |
| RM+ 10 g/L acetic acid | 3.77 |
| RM+ 11 g/L acetic acid | 3.74 |
| RM+ 12 g/L acetic acid | 3.71 |
| RM+ 13 g/L acetic acid | 3.67 |
| RM+ 14 g/L acetic acid | 3.64 |
| RM+ 15 g/L acetic acid | 3.61 |
| RM+ 150 mM sodium acetate* | 5.97 |
| RM+ 200mM sodium acetate* | 6.02 |
| RM+ 250mM sodium acetate* | 6.00 |

*Sodium acetate trihydratewas used, equaling to 12.3 g/L, 16.4 g/L and 20.5 g/L sodium acetate, respectively.

Figure S1


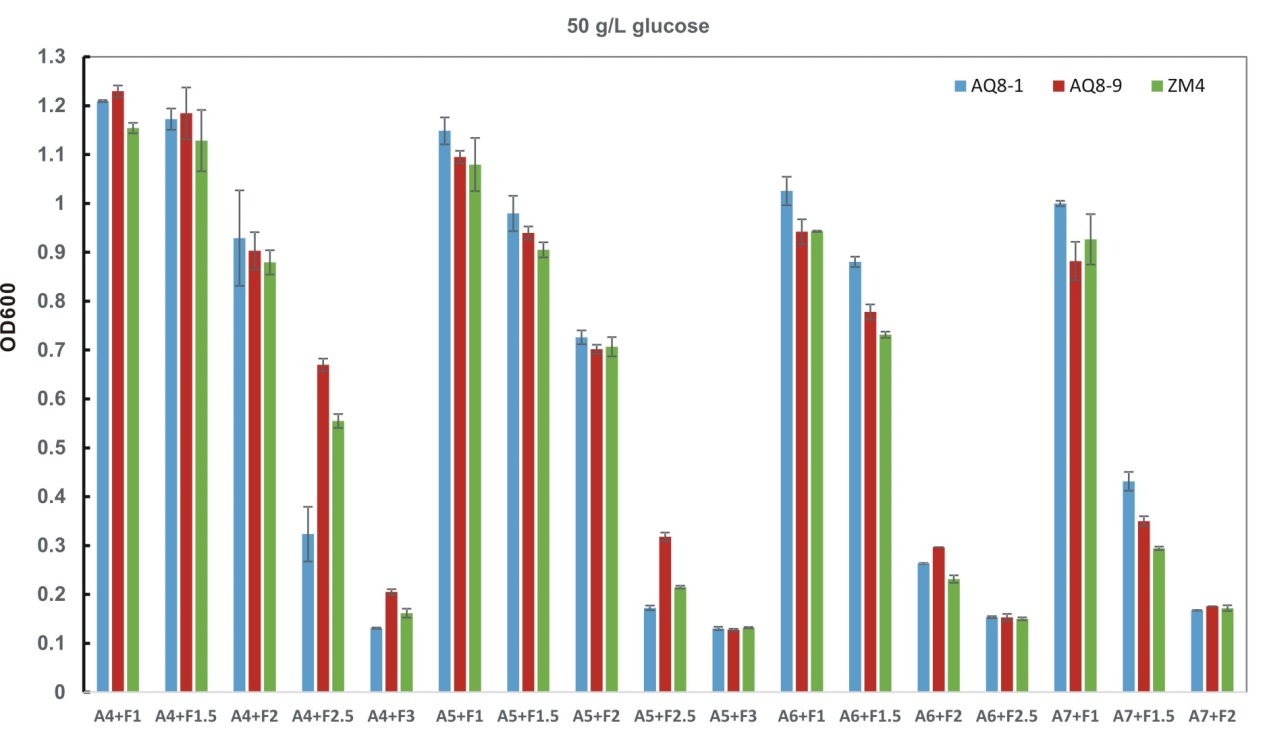


Tolerance of AQ8-1, AC8-9 and ZM4 to mixture of acetic acid and furfural. “A” indicates acetic acid and “F” indicates furfural. The number after A or F indicates the concentration of corresponding inhibitor. The concentrations of acetic acid ranged from 1.0 to 7.0 g/L, and furfural from 1.0 to 3.0 g/L in the mixture. OD_600 nm_ valueswere measuredat stationery phase.
